# Rhabdomyolysis plus Hypocalcemia and Diabetic Ketoacidosis as Concurrent Rare COVID-19 Manifestations

**DOI:** 10.1155/2021/6625086

**Published:** 2021-03-08

**Authors:** Maryam Heidarpour, Mehrbod Vakhshoori, Mohammad Ali Haghighatpanah, Leila Ashrafi, Farzin Khorvash, Bijan Iraj

**Affiliations:** ^1^Isfahan Endocrine and Metabolism Research Center, Isfahan University of Medical Sciences, Isfahan, Iran; ^2^Heart Failure Research Center, Cardiovascular Research Institute, Isfahan University of Medical Sciences, Isfahan, Iran; ^3^Isfahan University of Medical Sciences, Isfahan, Iran; ^4^Acquired Immunodeficiency Research Center, Isfahan University of Medical Sciences, Isfahan, Iran

## Abstract

**Background:**

Common manifestations of coronavirus disease 2019 (COVID-19) from its initial official introduction are mostly related to the respiratory system. However, other rarer presentations are reported nowadays. *Case Presentations*. We reported three cases of COVID-19-infected patients with rhabdomyolysis as well as two other rarer simultaneous signs, including hypocalcemia (Case 1) and diabetic ketoacidosis (DKA) (Case 2).

**Conclusion:**

Despite the fact that rhabdomyolysis is an infrequent manifestation of COVID-19, high clinical suspicion is required for proper diagnosis and management of this disease as well as other concurrent rarer presentations, including hypocalcemia and DKA for the prevention of further complications.

## 1. Introduction

Coronavirus disease 2019 (COVID-19) emerges as a novel infectious agent with several body organs' involvement. This disease's most common presentations are fever, dry cough, fatigue, or myalgia [1]. However, some patients develop severe symptoms, including shortness of breath, requiring further therapeutic support. On the other hand, rarer manifestations of COVID-19 have been declared.

Rhabdomyolysis is defined as muscle cell destruction resulting in subsequent leakage of cellular components to the bloodstream. This disease is mostly manifested with weakness, myalgia, electrolyte imbalance, myoglobinuria, or even acute kidney injury [2]. However, the exact etiology of rhabdomyolysis among COVID-19 patients needs to be investigated in future studies.

Moreover, hypocalcemia is a common phenomenon among critically ill patients, and it has been reported that calcium plays a pivotal role in the replication mechanisms of some viruses, including severe acute respiratory syndrome (SARS), Middle East respiratory syndrome (MERS), and Ebolavirus [3, 4]. For instance, a study done on patients suffering from SARS reported that hypocalcemia was prevalent in 70% of individuals during the hospitalization duration [4]. Another study investigated the prevalence of hypocalcemia among severely ill COVID-19 patients and figured out that almost two-thirds of them had lower calcium levels and had subsequent worse outcomes [5].

Diabetic ketoacidosis (DKA) is another rare manifestation during the COVID-19 pandemic. Goldman et al. reported that this disorder is prevalent among 1.8% of admitted patients [6]. Chan et al. performed a case series on six patients with COVID-19 admitted with DKA and/or hyperosmolar hyperglycemic state (HHS) and found that these two glucose imbalance disorders were the primary manifestations in most cases [7]. However, the data are limited, and several future studies in terms of predisposing factors are required.

Herein, we reported three cases of COVID-19 patients with rhabdomyolysis as well as other concurrent rarer symptoms, including hypocalcemia and DKA.

## 2. Case Presentation

### 2.1. Case 1

**Figure 1:**
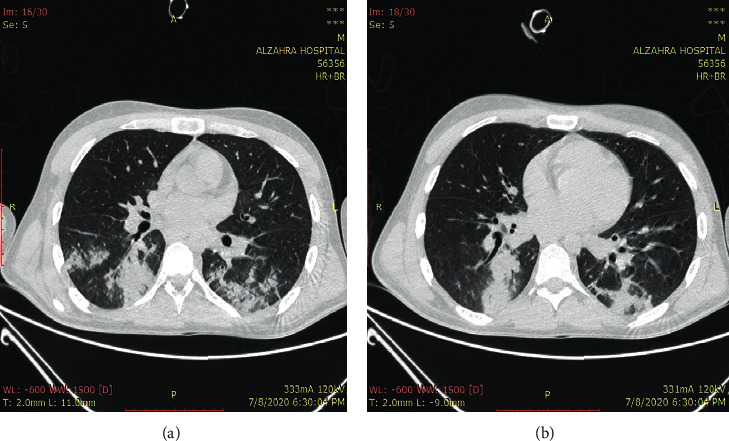
Multifocal peribronchial consolidation and diffuse ground-glass opacities.

A 22-year-old man with no previous past medical history was referred to the emergency department with severe hypocalcemia (Ca: 5 mg/dl) as well as spastic muscles in both upper and lower extremities. Also, he was opium-addicted and used 15 cc of daily methadone. He had severe bloodless diarrhea from 20 days before admission managed with serum therapy. His vital signs were in normal ranges. He was conscious, but he appeared to be pale. He did not have any positive history in terms of nausea, vomiting, cough, or dyspnea. However, he suffered from generalized muscle spasms and pain. There was not any positive finding during heart and lung examinations. His abdomen was nontender, but pitting edema (2+) was observed in the lower extremities. Chvostek's and Trousseau's signs were positive, and his electrocardiogram (ECG) revealed a prolonged QT interval (QT = 550 msec). Due to the high prevalence of COVID-19, lung high-resolution computed tomography (HRCT) was performed and the results were highly suggestive of COVID-19 infection (Figure 1).

In the first hour of admission, intravenous infusion of 10% calcium gluconate was initiated promptly, and muscle spasms disappeared within four hours. Intravenous calcium administration was then continued based on plasma-calcium and ECG monitoring. The patient had a myoclonic-like seizure with an upward gaze on the first night of hospitalization, while the serum level of calcium was 8.9 mg/dl. The result of the requested brain CT was negative. Unfortunately, the seizure recurred, and intravenous diazepam was prescribed. On the third day of admission, the calcium concentration reached 9.1 mg/dl, and the QT interval was corrected (QT = 405 msec). Other laboratory data collected during the admission time were as follows: lactic acid dehydrogenase (LDH) of 1740 U/L, creatine phosphokinase (CPK) of 1840 U/L, potassium of 6.9 mEq/l, sodium of 142 mEq/l, blood sugar of 234 mg/dl, albumin of 3.2 g/dl, aspartate aminotransferase (AST) of 261 IU/L, alanine aminotransferase (ALT) of 110 IU/L, alkaline phosphatase (ALP) of 379 IU/L, parathyroid hormone (PTH) of 145 pg/ml, 25-hydroxy vitamin D of 32 ng/mL, blood urea nitrogen (BUN) of 19 mg/dl, and creatinine (Cr) of 1.2 mg/dl.

His laboratory findings favored rhabdomyolysis, and fluid therapy was started promptly with concurrent seizure as well as hypocalcemia management to avoid acute kidney injury. Unfortunately, the patient had a cardiac and respiratory arrest, and cardiopulmonary resuscitation (CPR) was implemented, and he was connected to a ventilator. However, on the fourth day of hospitalization, the patient could not be resuscitated after the second cardiac arrest attack.

### 2.2. Case 2

**Figure 2:**
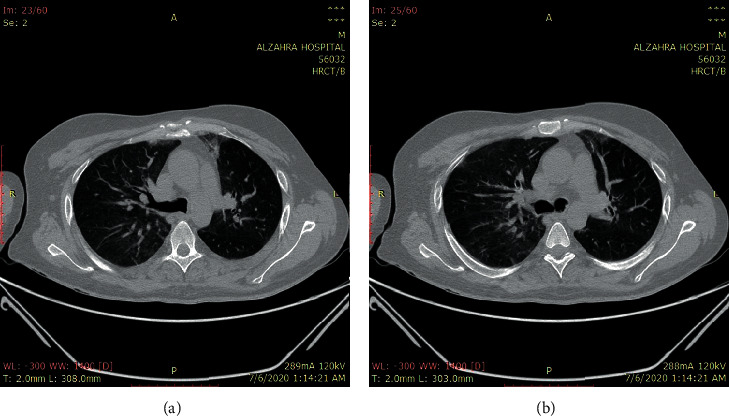
Bilateral and peripheral ground-glass pulmonary opacities.

A 36-year-old man was referred to the emergency department with two days of polyuria, polydipsia, nausea, and vomiting. His medical history was positive for psychosis, and he consumed olanzapine, lorazepam, propranolol, and sertraline for the last ten years. He was opium-addicted and used 20 cc of daily methadone. At admission, he was confused and lethargic with normal vital signs except for high temperature (*T* = 38.5°C). Besides dehydration, other physical examinations did not reveal any positive findings. His blood sample was taken and showed a blood sugar of 500 mg/dl, pH of 7, and HCO_3_ of 2.5 mEq/l. With the assumed diagnosis of DKA, insulin, tazocin, and serum therapy was initiated. The patient's general condition and blood sugar levels were improved during the next day, and the HCO_3_ concentration was raised to 9.5 mEq/l. On the third day of admission, his HCO_3_ was 11 mEq/l. However, insulin infusion was held due to a lower potassium level. The patient suffered from dyspnea and shortness of breath on the next day and was connected to the ventilator. HRCT was requested, and the results were highly suggestive of COVID-19 infection (Figure 2). Additional laboratory data were as follows: potassium of 7 mEq/l, sodium of 138 mEq/l, LDH of 1296 U/L, CPK of 5130 U/L, phosphorus of 2 mg/dl, and Cr of 5.3 mg/dl.

**Figure 3:**
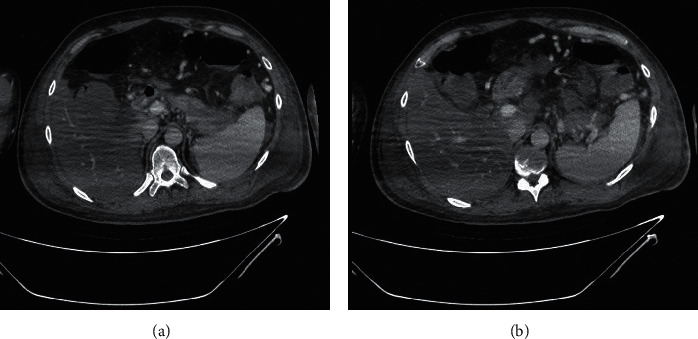
Heterogeneity and edema of the pancreatic tail with low enhancement (a) and splenic vein thrombosis with multiple collaterals (b).

Prompt therapeutic measurements were done, and because of decreased urine output, a temporary dialysis catheter was inserted, and he underwent hemodialysis. During the hospital admission, he experienced an attack of hypotension managed with norepinephrine as well as hydrocortisone. An abdominal computed tomography (CT) scan was performed due to increased amylase (142 U/L) and lipase (351 U/L) levels. The findings of imaging studies and the analysis of ascites fluid favored pancreatitis (Figure 3(a)). Moreover, this CT was highly suggestive of concurrent splenic vein thrombosis (Figure 3(b)). All appropriate therapies were implemented, and most of the laboratory data were improved consequently. The hemodialysis course was discontinued, and he was discharged from the hospital after the second abdominal CT examination indicated resolved signs of pancreatitis. On the 45th day, his general condition was good. He received insulin twice a day and daily orally administered warfarin with a dosage of 5 mg adjusted to keep the patient's international normalized ratio (INR) value of over 2.

### 2.3. Case 3

**Figure 4:**
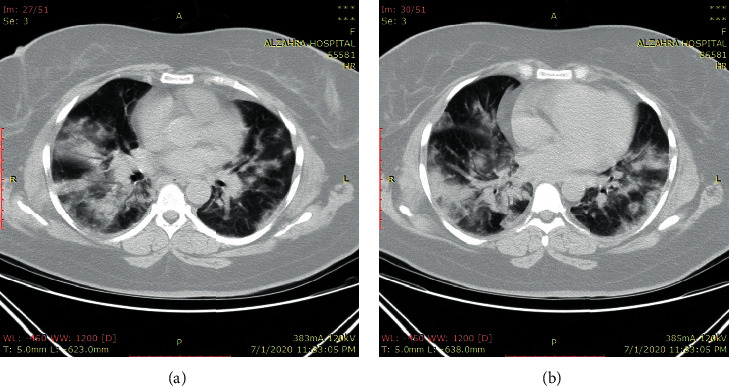
Multifocal multilobular consolidation and ground-glass opacities.

A 47-year-old woman came to the hospital with respiratory symptoms, including fever, cough, and dyspnea, for two weeks. She had a long history of diabetes mellitus, hypertension, hypothyroidism, and asthma, which had been medically controlled. Other vital signs were in normal ranges. She did not complain of muscle pain. Due to high suspicion for COVID-19 infection, she was tested, and the results of reverse transcriptase-polymerase chain reaction (RT-PCR) and HRCT were suggestive of this viral infection (Figure 4). She was transferred to the infectious ward. Her laboratory findings showed LDH of 5580 U/L, CPK of 16480 U/L, Cr of 1.7 mg/dl, AST of 951 IU/L, ALT of 874 IU/L, and ALP of 394 IU/L. With the assumed diagnosis of rhabdomyolysis, serum therapy was prescribed. The shortness of breath worsened on the fifth day and resulted in the New York Heart Association (NYHA) class of IV worsening cardiac function with severe hemoglobin oxygen desaturation due to bronchial secretions. Despite receiving 100% oxygen via a face mask, oxygen saturation only reached 80%. Therefore, the decision was made in order to intubate the patient. She was transferred to the intensive care unit (ICU), and the proper treatments were continued. Her laboratory findings were improved during the next week (LDH: 1204 U/L, CPK: 560 U/L, Cr: 0.7 mg/dl, AST: 135 IU/L, ALT: 234 IU/L, and ALP: 562 IU/L). The patient remained stable hemodynamically; however, she became unable to wean from a ventilator secondary to probable acute respiratory distress syndrome (ARDS). Unfortunately, in the third week, she had a sudden cardiac arrest. She underwent cardiorespiratory resuscitation but could not be resuscitated from her pulseless electrical activity arrest and died.

## 3. Discussion

Since the emergence of COVID-19, several other manifestations rather than pulmonary symptoms have been reported. Here, we presented three cases of confirmed COVID-19 infection with concomitant rhabdomyolysis as well as hypocalcemia and DKA. Rhabdomyolysis is a life-threatening syndrome characterized by skeletal muscle cell breakdown and leakage of cellular components, including potassium, phosphorus, CPK, and myoglobin, to the bloodstream. The typical triad of this disease consisted of myalgia, weakness, and dark-colored urine. However, less than 10% of patients experienced the classic triad [8]. Multiple etiologies have been suggested, including drug side effects, trauma, autoimmune status, exertion, ischemia, and infection [9]. Common viral causes include influenza A and B, herpes simplex virus, Epstein–Barr virus, cytomegalovirus, human immunodeficiency virus, and enteroviruses [10]. Even though our patients were not tested for other viral diseases, clinical status and laboratory data confirmed COVID-19 infection. While the exact pathophysiological mechanism attributed to rhabdomyolysis occurrence in COVID-19 infection has yet to be investigated, several theories have been declared. Due to the genomic similarity between SARS and COVID-19 and the ability of SARS to directly infect muscle cells, it probably seems that this new infective agent can infect myocytes. The formation of immune complexes and their deposition because of cross-reactivity between muscle cells and viral antigens might be responsible for muscular damage. Massive cytokine production and cytotoxic T-cell activation could be categorized as other possible causes in this regard [10, 11]. On the other hand, other rare comanifestations of COVID-19-induced rhabdomyolysis must be considered. Case 1 was admitted with severe hypocalcemia as the initial presentation. Although the patient had myalgia and experienced seizure, which might be due to rhabdomyolysis complications, the direct virus effect for causing these presentations should be considered. Liu et al. performed a retrospective study to assess the predictive effect of hypocalcemia among patients with severe COVID-19. They figured out that 62.6% of patients had low calcium levels, which was associated with poorer outcomes [5]. Hypocalcemia is quite common in viral infections and among critically ill patients. The possible factors include malnutrition, decreased intestinal absorption of calcium, cell membrane damage due to hypoxia and calcium influx, vitamin D deficiency, or medication interaction. Moreover, serum calcium is mainly bound to albumin, and hypocalcemia could occur in favor of reduced albumin levels. However, cytokine production during COVID-19 infection is reported to inhibit PTH secretion, and impaired response to PTH might be responsible for the induction of hypocalcemia [12]. Several studies are required to evaluate the exact pathophysiological mechanism responsible for hypocalcemia among COVID-19-infected individuals. Overall, in our case, it seems that impaired response to PTH, malnutrition, and calcium influx were possible causes of hypocalcemia.

In terms of DKA that occurred in Case 2, several points should be considered. The infection itself might be responsible for the induction of DKA [7]. Also, abnormal blood glucose has been reported to be associated with worse outcomes in the COVID-19 pandemic. Although the data are still limited, further investigations for the diagnosis of the exact mechanism are required. The Coronaviridae family has the tropism for both exocrine and endocrine pancreatic cells. Like SARS, angiotensin-converting enzyme 2 (ACE2) has been suggested to be a receptor for COVID-19 S-protein [13]. This receptor has been expressed in pancreatic beta-cells in addition to pulmonary cells [14]. The primary role of ACE2 is the degradation of angiotensin 2 to angiotensin1–7. Therefore, downregulation of the ACE2 receptor during COVID-19 leads to increased angiotensin 2 concentration resulting in reduced blood supply to pancreatic cells and subsequently delayed insulin secretion, which ultimately results in hyperglycemia and even DKA occurrence [14, 15]. However, whether the sudden onset alterations of glucose metabolism in severe COVID-19 will be persisted or remitted is still unclear. The presence of this receptor on the exocrine pancreas might explain the incidence of pancreatitis in Case 2. It seems that pancreatic involvement during COVID-19 should be further addressed in clinical settings.

In conclusion, our findings from this case report indicate that rhabdomyolysis could be categorized as one of the complications of COVID-19, and further suspicion for proper diagnosis and therapeutic interventions should be performed. Furthermore, other simultaneous rare manifestations, including hypocalcemia or DKA, must be comprehensively addressed in future studies.

## Data Availability

The datasets generated and/or analyzed during the current study are not publicly available due to confidential issues but are available from the corresponding author on reasonable request.

## Abbreviations

COVID-19: Coronavirus disease 2019
SARS: Severe acute respiratory syndrome
MERS: Middle East respiratory syndrome
DKA: Diabetic ketoacidosis
HHS: Hyperosmolar hyperglycemic state
ECG: Electrocardiogram
HRCT: High-resolution computed tomography
LDH: Lactic acid dehydrogenase
CPK: Creatine phosphokinase
AST: Aspartate aminotransferase
ALT: Alanine aminotransferase
ALP: Alkaline phosphatase
PTH: Parathyroid hormone
BUN: Blood urea nitrogen
Cr: Creatinine
CPR: Cardiopulmonary resuscitation
CT: Computed tomography
INR: International normalized ratio
RT-PCR: Reverse transcriptase-polymerase chain reaction
NYHA: New York Heart Association
ICU: Intensive care unit
ARDS: Acute respiratory distress syndrome
ACE2: Angiotensin-converting enzyme 2.
